# Deep Learning and Machine Learning with Grid Search to Predict Later Occurrence of Breast Cancer Metastasis Using Clinical Data

**DOI:** 10.3390/jcm11195772

**Published:** 2022-09-29

**Authors:** Xia Jiang, Chuhan Xu

**Affiliations:** Department of Biomedical Informatics, SOM, University of Pittsburgh, Pittsburgh, PA 15217, USA

**Keywords:** deep learning, DNN, machine learning, breast cancer, metastasis, metastatic breast cancer, prediction, non-image, clinical, EHR

## Abstract

Background: It is important to be able to predict, for each individual patient, the likelihood of later metastatic occurrence, because the prediction can guide treatment plans tailored to a specific patient to prevent metastasis and to help avoid under-treatment or over-treatment. Deep neural network (DNN) learning, commonly referred to as deep learning, has become popular due to its success in image detection and prediction, but questions such as whether deep learning outperforms other machine learning methods when using non-image clinical data remain unanswered. Grid search has been introduced to deep learning hyperparameter tuning for the purpose of improving its prediction performance, but the effect of grid search on other machine learning methods are under-studied. In this research, we take the empirical approach to study the performance of deep learning and other machine learning methods when using non-image clinical data to predict the occurrence of breast cancer metastasis (BCM) 5, 10, or 15 years after the initial treatment. We developed prediction models using the deep feedforward neural network (DFNN) methods, as well as models using nine other machine learning methods, including naïve Bayes (NB), logistic regression (LR), support vector machine (SVM), LASSO, decision tree (DT), k-nearest neighbor (KNN), random forest (RF), AdaBoost (ADB), and XGBoost (XGB). We used grid search to tune hyperparameters for all methods. We then compared our feedforward deep learning models to the models trained using the nine other machine learning methods. Results: Based on the mean test AUC (Area under the ROC Curve) results, DFNN ranks 6th, 4th, and 3rd when predicting 5-year, 10-year, and 15-year BCM, respectively, out of 10 methods. The top performing methods in predicting 5-year BCM are XGB (1st), RF (2nd), and KNN (3rd). For predicting 10-year BCM, the top performers are XGB (1st), RF (2nd), and NB (3rd). Finally, for 15-year BCM, the top performers are SVM (1st), LR and LASSO (tied for 2nd), and DFNN (3rd). The ensemble methods RF and XGB outperform other methods when data are less balanced, while SVM, LR, LASSO, and DFNN outperform other methods when data are more balanced. Our statistical testing results show that at a significance level of 0.05, DFNN overall performs comparably to other machine learning methods when predicting 5-year, 10-year, and 15-year BCM. Conclusions: Our results show that deep learning with grid search overall performs at least as well as other machine learning methods when using non-image clinical data. It is interesting to note that some of the other machine learning methods, such as XGB, RF, and SVM, are very strong competitors of DFNN when incorporating grid search. It is also worth noting that the computation time required to do grid search with DFNN is much more than that required to do grid search with the other nine machine learning methods.

## 1. Background

In 2020, female breast cancer surpassed lung cancer as the most commonly diagnosed cancer worldwide, with an estimated 2.3 million new cases in 2020 [1]. Breast cancer remains one of main cancer-related causes of death in women globally [2] and was responsible for 685,000 deaths worldwide in 2020 [1]. Breast cancer is the second leading cause of cancer death among US women after lung cancer, estimated to account for 43,600 deaths in 2021 [3,4,5]. It is the number one cause of cancer-related deaths for US women aged 20 to 59 [6].

Women rarely die of breast cancer confined to the breast or draining lymph nodes; rather, they die mainly due to metastasis, a condition in which cancer spreads to other vital organs, such as the lung and brain. Metastatic breast cancer (MBC) is the cause of over 90% of breast cancer related deaths [7] and remains a largely incurable disease. Although most newly diagnosed breast cancer cases are not metastatic, all patients are at risk of developing metastatic cancer in the future, even if they are free of cancer for years after the initial treatment. The ability to effectively predict, for each individual patient, the likelihood of later metastatic occurrence is important, because the prediction can guide treatment plans tailored to a specific patient to prevent metastasis and to help avoid under- or over-treatment.

Clinicians face uncertainty in determining the ideal treatment course for individual patients with breast cancer. For example, image-guided core needle biopsy of the breast is a common procedure that can return non-definitive results in 5% to 15% of women. In these cases, it is difficult to determine the subtype of the breast cancer. Variation in breast cancer subtypes has been known to be associated with a patient’s drug response, progression of the tumor, and survival of the patient [8,9]. There can also be significant uncertainty about the treatment and prognosis for breast cancer. For example, HER2-amplified breast cancer is a subtype with poor prognosis if untreated, but targeted therapeutic trastuzumab (Herceptin) has vastly improved the survival rate of such patients. Although Herceptin is used in the therapy of all patients with HER2-amplified tumors, only some respond. Furthermore, it is expensive and can cause cardiac toxicity [10]. Therefore, it is important to limit its usage to patients who are likely to benefit from it. Furthermore, histology alone does not predict long term outcome well, as most breast cancers are considered localized to the breast at the time of diagnosis, with most of these patients ‘cured’ upon excision. Still, up to one third of these patients will suffer distant recurrences, often after many years [11]. As treatments are toxic, clinical decisions need to account for prognostic predictors of outcome.

Various learning methods have been developed and applied in biomedical prediction [12,13,14,15,16,17,18]. For instance, machine learning and language processing have been used to identify breast cancer local recurrence [12]. A logistic regression model was developed for cancer classification and prediction [13]. Various machine learning methods were used for predicting ubiquitination sites by training models from physicochemical properties of protein sequences data [14]. Bayesian network learning was used to model miRNA–mRNA interactions that cause phenotypic abnormality in breast cancer patients [15]. The risk prediction of prostate cancer recurrence was investigated through regularized rank estimation in partly linear AFT (accelerated failure time) models using high-dimensional gene and clinical data [16]. An automatically derived class predictor was presented to determine the class of new leukemia cases based on gene expression monitoring by DNA micro-arrays [17]. An effective hybrid approach for selecting marker genes was developed for phenotype classification using micro-array gene expression data [18].

A neural network (NN) is one of the machine learning methods that can be used to conduct prediction and classification. A NN consists of layers of artificial neurons, also called nodes, structurally mimicking, in a sense, the impulse propagation mechanism in the human nervous system [19,20], so it is also called an artificial neural network (ANN). The traditional ANNs contain three layers arranged in a feedforward manner: an input layer, a hidden layer, and an output layer. Another type of neural network called multilayer perceptron (MLP) closely resembles the traditional ANNs structure-wise. ANNs can be used for unsupervised learning on unlabeled data or supervised learning on labeled data. Deep learning is the use of neural networks composed of more than one hidden layer, which are also referred to as deep neural networks (DNNs) [21,22,23].

Artificial neural networks (ANNs), including DNNs, are widely used in science and information technology due to their notable properties, including parallelism, distributed storage, and adaptive self-learning capability [24,25,26,27,28,29]. They have also been used in health care, including cancer diagnosis and prediction. For example, an ANN was developed to help diagnose breast cancer based on the age of the patient, mass shape, mass border, and mass density; it achieved high predictive accuracy [29]. A noise-injected neural network was designed for breast cancer diagnosis and prognosis using expression data [29]. A hybrid neural network and genetic algorithm method was applied to breast cancer detection [26]. In another study, an ANN was used to reduce the number of gene signatures for the classification of breast cancer patients and the prediction of clinical outcomes, including the capability to accurately predict breast cancer metastases [25]. The DNN has obtained significant success in commercialized applications, such as voice and pattern recognition, computer vision, and image processing [27,30,31,32,33,34,35]. However, its power has not been fully explored or demonstrated in clinical applications, such as the prediction of breast cancer metastasis (BCM). This is because the sheer magnitude of the number of variables involved in these problems presents formidable computational and modeling challenges [36].

Precision medicine promises to help us improve patient outcomes by tailoring healthcare to the individual patient [37]. The electronic health record (EHR), a widely available data resource, has been underutilized for the purpose of tailoring therapies and providing prognostic information. An EHR database contains abundant data about patients’ clinical features, disease status, interventions, and clinical outcomes, affording us the opportunity to provide highly personalized medicine beyond only looking at the genomic level. It is believed that “coupled with new analytics tools, they open the door to mining information for the most effective outcomes across large populations” [10]. Such data are invaluable to tailoring diagnosis and prognoses to individual with diseases such as breast cancer. 

The LSDS (Lynn Sage Dataset) was a de-identified and publicly available clinical dataset about breast cancer that was created and published via previous studies [38,39]. It was curated using clinical data from the Lynn Sage Database (LSDB) hosted at Lynn Sage Comprehensive Breast Center at Northwestern Memorial Hospital and the EHR data hosted at The Northwestern Medicine Enterprise Data Warehouse (NMEDW), Northwestern University, Feinberg School of Medicine and Northwestern Memorial HealthCare. The LSDS consists of records on 6726 breast cancer patients, which span 03/02/1990 to 07/28/2015. The dataset contains 61 patient features, including breast cancer metastasis and its follow-up [38,39]. Three LSM (LSDS for Metastasis) datasets were retrieved from LSDS, which focus on 5-, 10-, and 15-year BCM status, respectively [38,39]. A detailed description of the three LSM datasets are presented in the Methods Section.

In this research, we took the empirical approach to study the performance of DFNN models when predicting BCM using clinical data. Note that we describe the structure of our feedforward deep learning models in detail later in the Methods Section, and we refer to these models as the DFNN (deep feedforward neural network) models throughout the text. We applied DFNN method to learn prediction models from LSM datasets. These models can be used to predict 5-, 10-, and 15-year BCM. The performance of a DFNN model is affected by the number of hidden layers and number of nodes per hidden layer, which are called hyperparameters. In addition, there are other hyperparameters that can be used to adjust the prediction performance of deep learning. For example, “epochs” is a hyperparameter we consider. One epoch means a deep learning model is trained by each of the training set samples exactly once. The learning might not converge when epochs is too low, and model overfitting tends to get severe when it is too high. Tuning hyperparameters is the process of identifying the set of hyperparameter values that are expected to produce the best prediction model out of all sets of hyperparameter values examined. Grid search is designed to conduct hyperparameter tuning in a systematic way by going through a possible set of hyperparameter values automatically during learning. In this study, we optimized DFNN model performance by conducting hyperparameter tuning via grid search. 

To evaluate the performance of the DFNN, we compared our DFNN models with the ones that we trained using nine other well-known machine learning methods. We applied hyperparameter tuning and grid search to optimize model performance for each of the nine comparison methods. We conjectured that the performance of our DFNN models with grid search would be comparable to that of other machine learning methods when predicting the binary status of BCM. We posit this conjecture, because deep learning is a very powerful tool for prediction and has been successful in other applications, such as image recognition [35,40,41,42,43,44,45,46,47,48]. In this study, we use the DFNN models to predict 5-, 10-, and 15-year BCM by learning from non-image clinical EHR data. Through literature searching, we found some deep learning related studies that use image data to predict BCM [41,42,43,44,45], but we have not found a study that resembles ours. 

## 2. Methods

### 2.1. Datasets

In this study, we used three LSM datasets about breast cancer metastasis: LSM-5Year, LSM-10Year, and LSM-15Year. Missing data were filled in using the nearest neighbor (NN) imputation algorithm [39]. Metastatic case counts of each of the three datasets are shown in Table 1. Each of the three datasets contains 32 variables: 31 predictors and the target variable “metastasis.” Using LSM-5Year as an example, as described in [39], the value “yes” was assigned to “metastasis” if the patient metastasized within 5 years of initial diagnosis, the value “no” to “metastasis” if it was known that the patient did not metastasize within 5 years. The 31 predictors are defined in Table S1. Our objective was to learn and optimize prediction models from LSM datasets using DFNN and 9 other machine learning methods, and then to compare the performance of these models.

### 2.2. Feedforward Neural Networks

**Figure 1:**
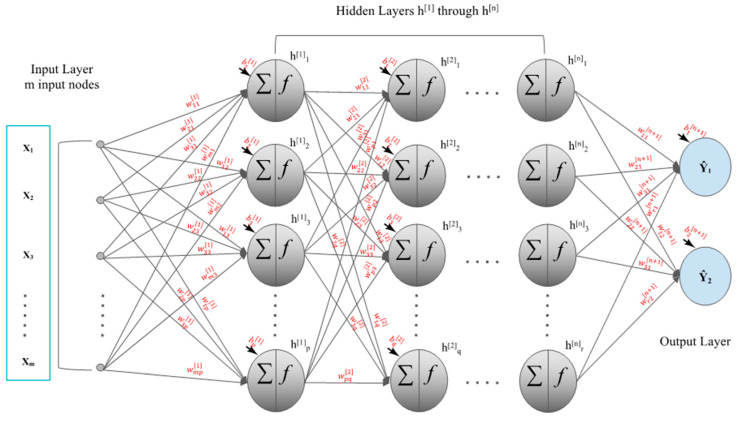
A DFNN (deep feedforward neural network) model that contains n hidden layers.

Our DFNN models are fully connected feedforward neural networks composed of more than one hidden layer. Figure 1 shows the general structure of a feedforward deep neural network that contains n hidden layers and an output layer that has two nodes. The inputs to the neural network are the observed values of the predictor variables in the dataset, while the outputs are the values of the target variable. In this research, we have 31 predictor variables, so m, the number of nodes in our input layer, is equal to 31. X_0_ represents the node for the bias passing from the input layer to the first hidden layer. The activation function $f\left(x\right)\;$of a node determines the value to be passed to the next node based on the value of the current node *x*. We used a rectifier linear unit (ReLU), in which $f\left(x\right)=\max\left(0,\;x\right)\;$, as the activation function in the input layer [36,37,38,39,40,41,42,43,44,45,46,47,48,49]. Since our datasets only contain positive values, by using ReLU as the activation function, all input values to our neural network model are directly passed to the hidden layers. In Figure 1, the first hidden layer has p hidden nodes, the second hidden layer has q hidden nodes, and the nth hidden layer has r hidden nodes, indicating each hidden layer is allowed to have a different number of hidden nodes. We used ReLU as the activation function in each of the hidden layer(s) to avoid the vanishing gradient problem [36,37,38,39,40,41,42,43,44,45,46,47,48,49]. $w_{ij}^{\left[1\right]}$(i=0, 2,…, m;  j=1, 2, ⋯,p)  represents the connecting weights between the input layer and the first hidden layer, $w_{jk}^{\left[2\right]}$(j=0, 1, 2, ⋯, p;  k=1, 2, ⋯, q) represents the connecting weights between the first hidden layer and the second hidden layer, and $w_{st}^{\left[n+1\right]}$(s=0, 2, ⋯, r;  t=1, 2) represents the connecting weights between the last hidden layer and the output layer. n is the number of hidden layers. $b^{\left[1\right]}{}_{j}$(j=1, 2, ⋯, p) represents the biases of the nodes in the first hidden layer, $b^{\left[2\right]}{}_{k}$(k=1, 2, ⋯, q) represents the biases of the nodes in the second hidden layer, and $b^{\left[n+1\right]}{}_{t}$(t=1, 2) represents the biases of the nodes in the output layer. We have two nodes in the output layer, one for each target value. Recall that “metastasis” is our binary target variable, which has two values: “yes” or “no”. We used the binary cross-entropy loss function, and sigmoid activation function in the output layer [36,37,38,39,40,41,42,43,44,45,46,47,48,49]. In this study, the initial values of weights and bias are provided by the he_normal weight initializer [50]. He_normal draws samples from a truncated normal distribution centered on 0 with $stddev=sqrt\left(2\;/\;num\_in\right)\;$where num_in is the number of nodes in a layer [50]. TensorFlow [51] is an open-source library widely used for developing deep learning models. Keras is a high-level neural network API built on top of TensorFlow [52,53]. Our DFNN model learner was coded in Python and implemented using the Keras and TensorFlow packages. 

### 2.3. Hyperparameter Tuning with Grid Search 

Deep learning is a powerful machine learning method due to its large number of hyperparameters that can be optimized [40]. See Table 2 for the hyperparameters and their values that we tested when training our DFNN models. The number of hidden layers and number of hidden nodes are structural hyperparameters that greatly affect model performance, each of which can assume numerous different values. SGD (stochastic gradient descent) and AdaGrad (adaptive gradient descent) are two commonly used optimizers. SGD adjusts its learning rate via momentum and decay, the two other hyperparameters that can be tuned during training. AdaGrad adapts the learning rate to the parameters, conducting smaller-step updates for parameters linked to frequently appearing features, and larger-step updates for parameters linked to less frequent features. The learning rate is a hyperparameter that governs how big of a step it takes each time to update the internal model parameters (weights and biases) in response to the estimated error during the model training process. It is used by both the SGD and AdaGrad. The momentum, a moving average of the gradients, is integrated in SGD to help accelerate the convergence of training. The decay is an iteration-based decay factor that can be used to decrease learning rate in each epoch during the optimization process. It is a hyperparameter incorporated in both SGD and AdaGrad to help optimize model performance. The batch_size is also a hyperparameter in Keras, which controls the number of the training samples that are “fed” to the neural network before internal model parameters are updated. Other hyperparameters, including epochs, dropout rate, L1, and L2, will be discussed in the “Overfitting” subsection below. 

Hyperparameter tuning is the process of identifying the set of hyperparameter values that is expected to produce the best prediction model from all sets of hyperparameter values being examined. Hyperparameter tuning gives us the power to optimize model performance but tuning a large number of hyperparameters presents a major challenge in terms of computation time [36,53,54]. Grid search is designed to conduct hyperparameter tuning in a systematic way by going through each of the sets of hyperparameter values automatically during the model training process [55]. In addition to grid search, there are other approaches of hyperparameter tuning, including Bayesian optimization and genetic algorithm [56]. One of the advantages of grid search is that the hyperparameter settings are independent. This makes it suitable to conduct parallel computing. Bayesian optimization and genetic algorithm are both considered as a type of sequential optimization method, with which the existing results will influence the subsequent model hyperparameter selection [57]. In this research, we focus on testing our central hypothesis, which states that our feedforward deep learning model with grid search is a competitive machine learning method when predicting the binary status of BCM.

We tried to improve model performance by conducting grid search implemented in Python using the scikit-learn package [51,52]. In a grid search, each of the hyperparameters is given a series of values; the program will then iterate through every hyperparameter value combination possible to train models. We call a hyperparameter value combination a hyperparameter setting. We conducted grid search many times, each time focusing on giving a set of values to each of the hyperparameters. In this research, we took a heuristic approach to select the range of hyperparameter values that are fed to a grid search. The range of values for a hyperparameter are predetermined in various ways, such as preliminary experiments, literal searching, and computation resource and time limitation that we have. For example, we decided to focus on checking up to 4 hidden layer models, because we found that further extending number of hidden layers takes up too much computing power but with overall worse results based on some preliminary experiments we conducted. So, the deepest model we trained contains 6 layers, counting the input and output layer. Another example is that the model performance normally becomes worse once epochs exceeds 800 based on our preliminary experiments, so we set the maximum number of epochs to be 800. In each grid search, we randomly chose a set of values from the range of values for each of the hyperparameters (Table 2) based on the maximum number of hyperparameter settings that we can handle in reasonable time.

### 2.4. Overfitting

Overfitting is a phenomenon in which the model performs well on training data but generalizes poorly to unseen data [58,59,60]. Overfitting occurs when the model is complex and has a large number of parameters, such as in a DNN model, but insufficient data to accurately capture the underlying relationships between the variables [58]. Overfitting is a common problem in machine learning, and it is overwhelmingly discussed in deep learning due to its significant effect on the performance of DNN models. A Google search using “overfitting in deep learning” identified 280,000 articles published between 2015 and 2020. This is not only because we are dealing with a large set of hyperparameters in deep learning, but also because the number of internal parameters increases dramatically as the number of hidden layers and the number of hidden nodes per layer increase.

It is not possible to completely eliminate overfitting, but we took multiple approaches to minimize the effect of overfitting. First, we tuned “dropout rate” and “epochs” to reduce the effect of overfitting [40]. The “dropout” is a hyperparameter with which neurons are randomly dropped out during training to reduce time cost and minimize model overfitting [40]. The epochs is a hyperparameter that helps balance model convergence and overfitting [59]. It defines the number of times that the entire training data are used by the learning algorithm during training. One epoch means every sample in the training set has been used exactly once to update the internal model parameters. Secondly, we tuned regularization hyperparameters L1 and L2 to reduce overfitting. L1, a factor associated with LASSO regularization, can be used to remove the effect of the “noisy” input nodes and make the network less complex [60]. L1 is also called a sparsity regularization factor. L2 is a regularization factor based on weight-based decay, which penalizes large weights to adjust the weight updating step during model training [60]. We also introduced another parameter, named “L1OrL2”, with which we can choose to tune L1 alone, L2 alone, or L1 and L2 simultaneously in a grid search. Finally, we used percent_AUC_diff to quantify and keep track of the overfitting of a model. The percent_AUC_diff is an output parameter in our grid search procedure, which represents the percent difference between mean train AUC and mean test AUC. When we selected the best DFNN models, we not only considered the mean test AUC values, but also made sure the percent_AUC_diff was less than 5%.

#### 2.4.1. Performance Metrics and 5-Fold Cross-Validation

We designed an output format for grid search and recorded 64 different output values for each of the models trained in a grid search. Among the output values are information about the computer system used, computation time, and measures for model performance. For a given binary diagnostic test, a receiver operator characteristic (ROC) curve plots the true positive rate against the false positive rate for all possible cutoff values []. The area under an ROC curve (AUC) measures the discrimination performance of a model. We conducted a 5-fold cross-validation to train and evaluate each model in a grid search. The entire dataset was split evenly into 5 portions. The division was mostly executed randomly, except that each portion had approximately 20% of the positive cases and 20% of the negative cases to ensure that it was a representative fraction of the dataset. Training and testing were repeated five times. Each time, a unique portion was used as the validation set to test the model learned from the training set, which combined the remaining four portions. Training and testing AUCs were reported. The average training and testing AUC across all five times was also derived and reported. The best-performing set of hyperparameter values was chosen based on the highest mean test AUC. The best model would be the one refitted from the entire dataset using the best-performing set of hyperparameters values. We used this procedure for all methods involved in this study.

#### 2.4.2. Comparison to 9 Other Machine Learning Methods

We compared the performance of the best-performing DFNN model to that of a representative set of machine-learning methods, each obtained via grid search. The representative set of methods include naïve Bayes (NB), logistic regression (LR), decision tree (DT), support vector machine (SVM), the least absolute shrinkage and selection operator (LASSO), k-nearest neighbor (KNN), eXtreme gradient boosting (XGB), adaptive boosting (AdaBoost), and random forest (RF). We used the scikit-learn [51,52] package in Python to implement these machine learning classifiers. Like neural networks, these methods have hyperparameters that can be tuned to improve prediction performance. We conducted grid search for each method using each of the three LSM datasets. Like we did in our DFNN grid-searches, we conducted 5-fold cross-validation for each set of hyperparameter values and measured the performance by the AUC. Table 2 provides a summary of the hyperparameters and their values that we tested for each of these methods.

**NB** (naïve bayes) [61,62,63,64] represents a special type of Bayesian network model. Bayesian networks (BNs) are used for uncertain reasoning and machine learning in many domains, including biomedical informatics [62]. *A* BN (bayesian network) consists of a directed acyclic graph (DAG) $G=\left(V,\;E\right)$, whose nodeset *V* contains random variables and whose edges *E* represent relationships among the random variables [64]. A BN also includes a conditional probability distribution of each node *X∈ V* given each combination of values of its parent nodes. Each node *V* in a BN is conditionally independent of all its nondescendents given its parents in the BN. NB is a simplified BN which normally only contains one parent node and a set of children nodes. In a basic NB model, there is an edge from the parent to each of the children. When a NB model is used to conduct classification, it is called a NB classifier. We used the BernoulliNB classifier in this study because we have binary classes. Alpha is the Laplace smoothing parameter that deals with the problems of zero probability and regularize complexity, the larger the alpha, the stronger the smoothing and the lower the complexity of the model. We tested 500 alpha values, which are all positive integers from 1 to 500.

**LR** (logistic regression) [65,66] is a supervised learning classification method, which is normally suitable for binary classification problems. It is named after the logistic function, a core function of LR for nonlinear transformation on the output value [65]. *C* is the inverse of regularization strength *(*C=1/λ). Smaller values result in stronger regularization [66]. We tested 300 evenly spaced values on a logarithmic scale between $10^{-4}$ and $10^{4}.$ Regularization can be used to train models that generalize better on unseen data by preventing the algorithm from overfitting the training dataset [65,66]. We used either L1 or L2 methods to regularize the LR model.

**DT** (decision tree) [67,68] is one of the most widely used machine learning methods. It contains a tree-like structure in which each internal node represents a test on a feature and each leaf node represents a class value [67]. It can be used for both classification and regression tasks. This parameter max_depth indicates how deep the tree can be. The deeper the tree, the more splits it has, which allows it to capture more information about the data [68]. We fit a decision tree with depths ranging from 3 to 32. The parameter min_samples_split governs the minimum number of samples required to split an internal node. The values we tested in our grid search are 0.1, 0.2, 0.3, 0.4, 0.6, 0.8, and 1. The parameter max_features indicates the max features when building a decision tree; we tested all values: none, ‘log2′, and ‘sqrt’. The parameter max_leaf_nodes controls the maximum number of leaf nodes of each decision tree, we tested 7, 10, 15, and none. max_depth and max_leaf_nodes are important hyperparameters to control overfitting. Criterion is a function for measuring the quality of a split, and we tested both values ‘gini’ and ‘entropy’.

**SVM** (support vector machine) [69,70,71,72,73,74,75] is a machine learning method that identifies a hyperplane with margins defined by support vectors. Support vectors are a set of data points that are closer to the hyperplane and can influence both the position and direction of the hyperplane, which can be used to classify (separate) input samples. SVM can be used for both regression and classification tasks, and it is widely applied in the later [69,70,71]. The parameter C trades off misclassification of training examples against simplicity of the decision surface [72]. Smaller values result in a smoother decision surface, while larger values give the model more freedom to select more samples as support vectors [72,73,74,75]. We tested values in the range 2^−5^, 2^−3^… 2^15^. The parameter γ defines how far the influence of a single training example reaches (inverse of the radius of influence of samples selected by the model as support vectors). Low values mean “far” and high values mean “close”. We tested values in the range 2^−15^, 2^−13^… 2^3^.

**LASSO** (the least absolute shrinkage and selection operator) is a regression-based method classifier that is capable of conducting variable selection and regularization in order to enhance prediction performance and control overfitting [76]. The parameter alpha is the sum of absolute value of coefficients which provides a trade-off between balancing residual sum of squares and magnitude of coefficients. Alpha can take various values that are greater than 0. We tested 400 evenly spaced alpha values on a logarithmic scale between $10^{-5}$ and $10^{5}.$

**KNN** (k-nearest neighbor) [77,78,79] is a supervised machine learning method that can be used for both classification and regression tasks. KNN predicts the class value of an incoming sample by its k-nearest neighboring data points [77]. KNN assumes that cases with similar covariate values are near to each other. The parameter k_neighbors is the number of training samples closest in distance to a query point in order to predict the label of the query. We tested all integers between 1 and 300. The parameter weight is the weighting criteria used to assign a value to a query point [78,79]. We tested both the two available values uniform and distance. The value uniform assigns uniform weights to each neighbor. The value distance assigns weights to neighbors proportional to the inverse of the distance from the query point, so closer neighbors would weigh more. Metric is a parameter for choosing the method for calculating distance. We tested all available values, which are ‘eluclidean’, ‘manhattan’, and ‘chebyshev’.

**RF** (random forest) [68,80,81,82,83] is a typical model of bagging in ensemble learning, the trainer will randomly select a certain amount of sample data and create a corresponding decision tree. Many of these decision trees form a random forest [68,80]. An advantage of RF is that the independent character of each decision tree tends to reduce overfitting [81]. The parameter n_estimators is the number of decision trees in the random forest [83]. We tested values 10, 50, 60, 70,…, 200, and 500; Other parameters come from DT, and we tested the same values as we did with DT for them.

**ADB** (adaptive boosting) [82,83,84,85] is a typical model of boosting in ensemble learning. Unlike the RF model, where each decision tree is independent, AdaBoost is a classifier with cascade structure which means the next learner is based on the result of the previous weak learner [84]. During the learning process, if the current sample is classified incorrectly, the degree of difficulty of the sample will increase to make the next learner focus on the difficult part on which previous model performed poorly [85]. The parameter n_estimators is the number of weak learners. A model tends to overfit for large values of n_estimators. The values of n_estimators we tested include 10, 20, …, and 100. Learning_rate is used to shrink the contribution of each classifier. We tested all values from 0.002 to 0.01 with an increment of 0.001.

**XGB** (eXtreme gradient boosting) [86,87,88,89,90,91,92] is another common approach for boosting in ensemble learning. Unlike ADB, it uses gradient boosting. The XGB classifier is based on the difference between true and predicted values to improve model performance [86,87,88,89]. The parameter gamma is a pseudo-regularization hyperparameter in gradient boosting, and it affects pruning to control the overfitting problem [90,91,92]. Gamma values we tested are 0, 0.01, 0.1, 0.3, 0.5, and 0.9. The parameter min_child_weight is minimum sum of weights of all observations of a child node. The larger the value, the more conservative the algorithm will be. The values tested were 1, 2, 4, and 6. Alpha and lambda are both regularization hyperparameters which can help control overfitting. The values we tested for each of them are $1\times 10^{-5}$, 0.01, 0.1, 1, and 100. The parameter max_depth is the maximum depth of the individual regression estimators. The values of max_depth we tested were 3, 4, 5, …, 30, 31. The learning_rate values we tested were 0, 0.01, 0.1, 0.3, and 0.5.

#### 2.4.3. Statistical Testing

We conducted the Wilcoxon rank sum tests to determine the statistical significance of the AUC results. We conjectured that deep learning with grid search would perform comparably to other methods when predicting the binary status of 5-, 10-, and 15-year BCM. We paired the DFNN with each of the 9 other machine learning methods, and conducted both the right-tailed (greater) and left-tailed (less) Wilcoxon tests for each pair of the methods and repeated these tests for each of the three datasets separately. The null hypothesis for all the Wilcoxon tests is that the two methods perform indifferently. The alternative hypothesis of the right-tailed Wilcoxon tests is DFNN does better (greater) than the comparison method, and this is to test whether DFNN performs better than other methods. The alternative hypothesis of the left-tailed Wilcoxon tests is DFNN does worse (less) than other methods, and this is to test whether DFNN performs worse than the comparison method. We conducted the Wilcoxon rank sum test in R using the wilcox.test function included in the R package.

## 3. Results

Table 3 shows the mean AUCs from 5-fold cross-validation of the best-performing model for each method and each dataset, selected based the grid search results. Table S3 contains the results of the right-tailed Wilcoxon rank sum tests in which the alternative hypothesis is that the first method performs better (greater) than the second method in a pair of methods, while Table S4 shows the results of the tests in which the alternative hypothesis is the first method performs worse (less) than the second method. As shown in the first row of Table S3, X represents the first method and Y represents the second method. For example, in the cell of row 1 and column 2, DFNN is the first method and NB is the second method. Tables S3 and S4 include W, the *p*-value, and the 95% confidence interval (CI) for each of the Wilcoxon tests we conducted. W is the test statistic used in the Wilcoxon rank sum test.

Table 4 contains the hyperparameter values of the best-performing DFNN models learned from grid search using each of the three datasets. For example, the best model trained using the LSM-15year dataset contains 3 hidden layers, and each of them contains 300 hidden nodes; When we selected the best models, we not only considered the mean test AUC values, but also considered the percent_AUC_diff as defined previously. To identify the best-performing DFNN model, we first ordered the result table according to the mean test AUC values going from the highest to lowest. Then we looked at the percent_AUC_diff values from the top of the ordered results and selected as the best model the first model whose percent_AUC_diff value was less than 5%. Table 5 shows the average experiment time per model (in seconds), the number of all models trained via grid search, and total experiment time (in days) for each method and dataset.

**Figure 2:**
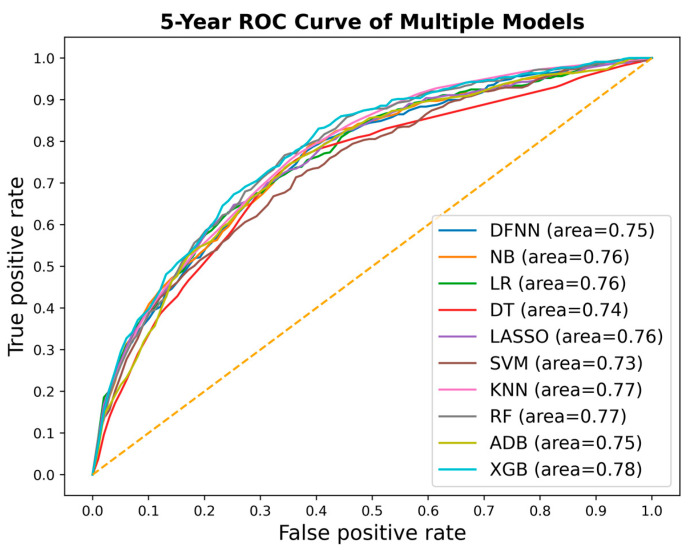
ROC curves of the best-performing models for all methods, each respectively, for predicting 5-year metastasis (ROC: receiver operating characteristic; DFNN: Deep feedforward neural network; NB: Naïve bayes; LR: Logistic regression; DT: Decision tree; SVM: Support vector machine; LASSO: Least absolute shrinkage and selection operator; KNN: K-nearest neighbor; RF: Random forest; ADB: AdaBoost; XGB: XGBoost).

**Figure 3:**
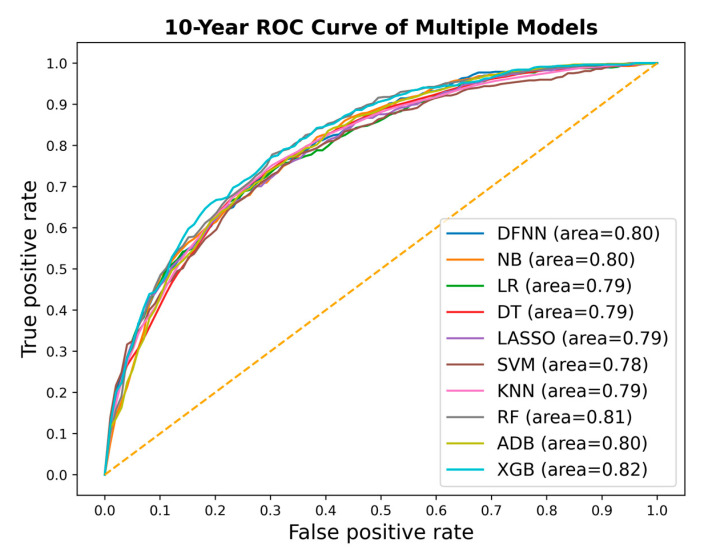
ROC curves of the best-performing models for all methods, each respectively, for predicting 10-year metastasis (ROC: receiver operating characteristic; DFNN: Deep feedforward neural network; NB: Naïve bayes; LR: Logistic regression; DT: Decision tree; SVM: Support vector machine; LASSO: Least absolute shrinkage and selection operator; KNN: K-nearest neighbor; RF: Random forest; ADB: AdaBoost; XGB: XGBoost).

**Figure 4:**
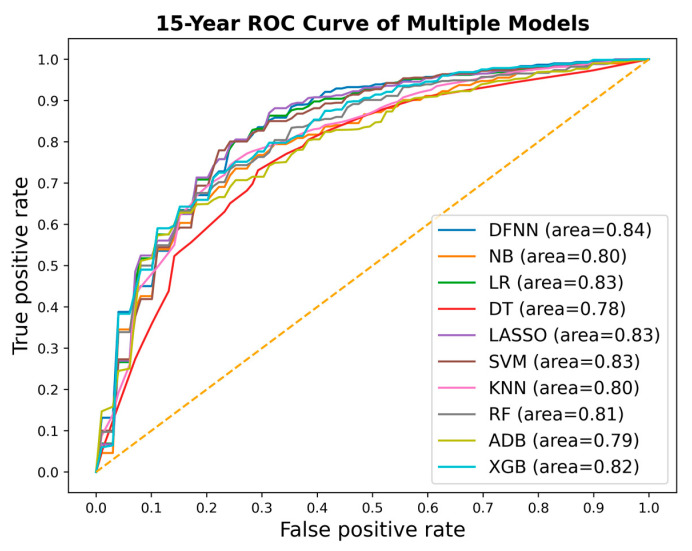
ROC curves of the best-performing models for all methods, each respectively, for predicting 15-year metastasis (ROC: receiver operating characteristic; DFNN: Deep feedforward neural network; NB: Naïve bayes; LR: Logistic regression; DT: Decision tree; SVM: Support vector machine; LASSO: Least absolute shrinkage and selection operator; KNN: K-nearest neighbor; RF: Random forest; ADB: AdaBoost; XGB: XGBoost).

**Figure 5:**
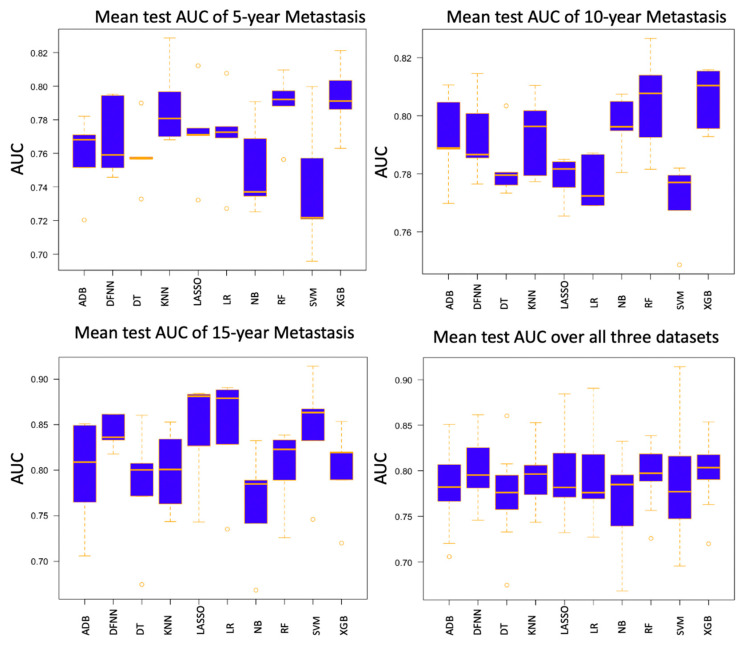
Boxplots to compare the mean test AUCs of all methods (AUC: area under the ROC curves; DFNN: Deep feedforward neural network; NB: Naïve bayes; LR: Logistic regression; DT: Decision tree; SVM: Support vector machine; LASSO: Least absolute shrinkage and selection operator; KNN: K-nearest neighbor; RF: Random forest; ADB: AdaBoost; XGB: XGBoost).

**Figure 6:**
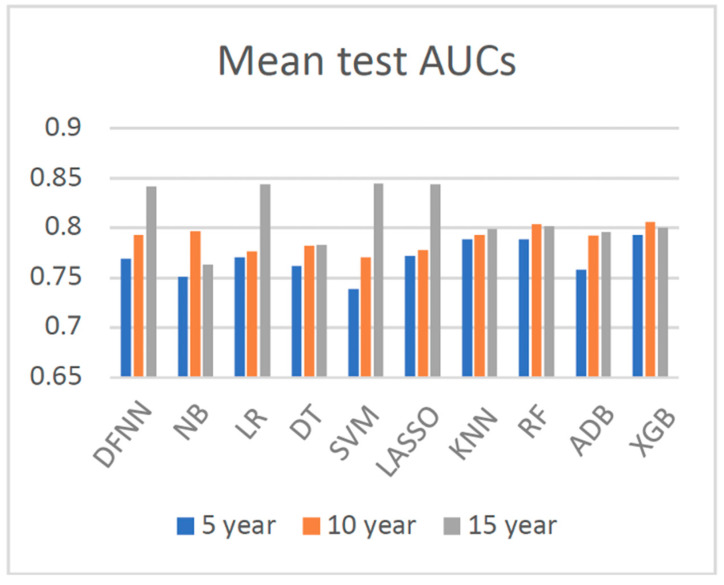
Side by side comparisons of the mean test AUCs of all methods when predicting 5-, 10-, and 15-year breast cancer metastasis (AUC: area under the ROC curves; DFNN: Deep feedforward neural network; NB: Naïve bayes; LR: Logistic regression; DT: Decision tree; SVM: Support vector machine; LASSO: Least absolute shrinkage and selection operator; KNN: K-nearest neighbor; RF: Random forest; ADB: AdaBoost; XGB: XGBoost).

We compared side by side the ROC curves of the best-performing models of DFNN and the nine comparison methods. Figure 2, Figure 3 and Figure 4 show these comparisons in the prediction of 5-, 10-, and 15-year BCM, each respectively. Figure 5 contains four panels of boxplots for comparing mean test AUC values of all methods side by side, one for each dataset separately and one for all datasets combined. We noticed that for each of the methods, including deep learning, the prediction performance improves in general as the number of years it takes to metastasize increases. We also notice that LR, LASSO, SVM, and DFNN perform extremely well when predicting 15-year BCM. We demonstrate this using a bar graph, as shown in Figure 6.

## 4. Discussion

Based on the mean test AUC values shown in Table 3, XGB (1st), RF (2nd), and KNN (3rd) are the top three methods in predicting 5-year BCM. DFNN ranks 6th and performs better than NB, DT, SVM, and ADB in this category. When predicting 10-year BCM, XGB (1st), RF (2nd), and NB (3rd) are the top three performers. DFNN and KNN tie as the number 4 performers, so DFNN performs better than LR, DT, SVM, LASSO, and ADB in this category. When predicting 15-year BCM, SVM (1st), LR and LASSO (tie for 2nd) and DFNN (3rd) are the top three performers, so in this category, DFNN outperforms the other six methods, including NB, DT, KNN, RF, ADB, and XGB.

We notice that in each of the three metastasis categories, the mean test AUC values of the top performers are quite close to each other. For instance, when predicting 15-year BCM, the mean test AUC values of the top four performers are 0.842 (DFNN), 0.844 (LR), 0.844 (LASSO), and 0.845 (SVM). We further look at the statistical testing results shown in Tables S3 and S4 to compare DFNN with each of the nine other machine learning methods. As shown in Table S3, the *p*-values we obtained for each pair methods range from 0.111 (DFNN vs. NB) to 0.925 (DFNN vs. KNN) in predicting 5-year BCM, which indicates that at a significance level of 0.05, we are not confident in rejecting the null hypothesis which states that DFNN performs no difference from the comparison methods. Table S3 also shows that DFNN performs better than both LASSO (*p*-value 0.028) and SVM (*p*-value 0.028), but no difference from other methods at a significance level of 0.05 when predicting 10-year BCM. Again, according to Table S3, DFNN performs better than NB, DT with a *p*-value of 0.011 and 0.030 each, respectively, but no difference from other methods at a significance level of 0.05 when predicting 15-year BCM. Based on Table S4, DFNN performs comparably to any of the other methods at a significance level of 0.05 for any of the three BCM categories. Overall, our statistical testing results support our conjecture that deep learning with grid search performs comparably to the other methods when predicting the binary status of BCM.

### 4.1. The Potential Effects of Data Imbalance

**Figure 7:**
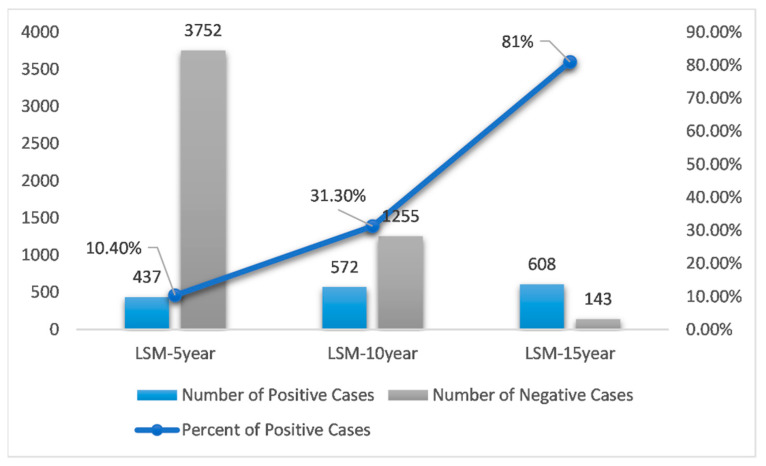
A comparison of the data imbalance status of LSM-5 year, 10 year, and 15 year datasets (LSM: Lynn Sage Dataset for Metastasis).

As demonstrated in Figure 6, the prediction performance of all methods improves in general as the number of years to metastasis increases. Data imbalance is normally referred to the situation when the number of positive cases and the number of negative cases is not equal when having a binary class. Although in many real datasets the negative cases are the majority ones, the positive cases are often more of research interests. Concurrently, as shown in Table 1 and Figure 7, the status of data imbalance has changed significantly as the number of years to metastasis increases. This may indicate that data imbalance, and more specifically in our case, the number of positive (metastasized) cases contained in the dataset has, in general, a positive effect on the prediction performance of these machine learning methods. Additionally, we observe that the mean test AUCs of DFNN, SVM, LASSO, and LR, when predicting the 15-year BCM, are significantly higher than that of these methods when predicting the 5-year and 10-year BCM. An explanation for this is the 15-year dataset has higher percentage of positive cases than the 5-year and 10-year datasets, as shown in Figure 7. This may indicate that these four methods are more sensitive to the percentage of positive cases contained in a dataset than other methods. The two ensemble methods XGB and RF outperform all other methods when predicting the 5-year and 10-year BCM, for which the positive class is the minority. This may indicate that these ensemble methods tend to handle this type of imbalanced data better.

### 4.2. Computation Time

Table 5 shows that the average experiment time per model of DFNN is much higher than that of any other method. This is perhaps because DFNN has a large number of hyperparameters, and its internal parameters (weights and biases) rapidly increase as the number of hidden nodes and the number of hidden layers are increased.

## 5. Conclusions

Based on the statistical testing results, we conclude that at a significance level of 0.05, DFNN performs comparably to any of the nine other methods when predicting 5-, 10-, and 15-year BCM. This is consistent with our conjecture that deep learning with grid search performs comparably to the other methods when predicting the binary status of BCM. On the other hand, it is interesting to learn that some of the other machine learning methods, such as XGB, RF, and SVM, are very strong competitors of DFNN. Additionally, obtaining the best-performing DFNN models required much more computation time than doing so for the nine comparison methods.

## Figures and Tables

**Table 1:** Case counts of the LSM datasets (#: number).

	Total # of Cases	# Positive Cases	# Negative Cases
LSM-5year	4189	437	3752
LSM-10year	1827	572	1255
LSM-15year	751	608	143

**Table 2:** Description of the DFNN (deep feedforward neural network) hyperparameters and their values tested (note: # represents the word “number” in this table).

Hyperparameter	Description	Values
# of Hidden Layers	The depth of a DFNN	1, 2, 3, 4
# of Hidden Nodes	Number of neurons in a hidden layer	10, 20, …, 70, 75, 80, 90, … 120, 200, 300, …, 1100
Optimizer	Optimizes internal model parameters towards minimizing the loss	SGD (stochastic gradient descent), AdaGrad
Learning rate	Used by both SGD and AdaGrad	0.001 to 0.3, step size: 0.001
Momentum	Smooths out the curve of gradients by moving average. Used by SGD.	0, 0.4, 0.5, 0.9
Iteration-based decay	Iteration-based decay; updating learning rate by a decreasing factor in each epoch	0 0.0001, 0.0002, …, 0.001, 0.002, …, 0.01
Dropout rate	Manage overfitting and training time by randomly selecting nodes to ignore	0, 0.4, 0.5
Epochs	Number of times model is trained by each of the training set samples exactly once	20, 30, 50, 80, 100, 200, …, 800
Batch_size	Unit number of samples fed to the optimizer before updating weights	1, 10, 20, …, 100
L1 (Lebesgue 1)	Sparsity regularization	0, 0.0005, 0.0008, 0.001, 0.002, 0.005, 0.008, 0.01, 0.02, 0.05, 0, 0.1, 0.2, 0.5
L2 (Lebesgue 2)	Weight decay regularization; it penalizes large weights to adjust the weight updating step	0, 0.0005, 0.0008, 0.001, 0.002, 0.005, 0.008, 0.01, 0.02, 0.05, 0, 0.1, 0.2, 0.5
L1ORL2	Using L1 and L2 combinations to regularize overfitting	L1 only, L2 only, L1 and L2

**Table 3:** The mean test AUCs and mean train AUCs of the best-performing models (LSM: Lynn Sage Dataset for Metastasis; DFNN: Deep feedforward neural network; NB: Naïve bayes; LR: Logistic regression; DT: Decision tree; SVM: Support vector machine; LASSO: Least absolute shrinkage and selection operator; KNN: K-nearest neighbor; RF: Random forest; ADB: AdaBoost; XGB: XGBoost).

Mean Test AUC/Mean Train AUC	LSM-5 Year	LSM-10 Year	LSM-15 Year
DFNN	0.769/0.806	0.793/0.830	0.842/0.873
NB	0.751/0.753	0.797/0.798	0.763/0.826
LR	0.771 /0.773	0.777/0.809	0.844/0.884
DT	0.762/0.780	0.783/0.827	0.783/0.838
SVM	0.739/0.811	0.771/0.808	0.845/0.867
LASSO	0.772/0.774	0.778/0.806	0.844/0.887
KNN	0.789/0.816	0.793/0.819	0.799/0.832
RF	0.789/0.801	0.804/0.840	0.802/0.849
ADB	0.759/0.754	0.792/0.800	0.796/0.829
XGB	0.793/0.813	0.806/0.845	0.800/0.854

**Table 4:** The hyperparameter values of the best-performing DFNN models learned from 5-year, 10-year, and 15-year datasets, respectively (LSM: Lynn Sage Dataset for Metastasis).

Hyperparameter Values of the Best-Performing Model	LSM-5 Year	LSM-10 Year	LSM-15 Year
Number of hidden layers.	2	1	3
Number of hidden nodes	{75, 75}	{75}	{300, 300, 300}
Kernel initializer	he_normal	he_normal	he_normal
Optimizer	SGD	SGD	SGD
Learning rate	0.005	0.01	0.005
Momentum Beta	0.9	0.9	0.9
Iteration-based decay	0.01	0.01	0.01
Dropout rate	0.5	0.5	0.5
Epochs	100	100	100
L1 (Lebesgue 1)	0	0	0
L2 (Lebesgue 1)	0.008	0.008	0.008
L1 and L2 combined	No	No	No

**Table 5:** Experiment time per model per dataset, number of models trained, and total experiment time. (#: number; LSM: Lynn Sage Dataset for Metastasis; DFNN: Deep feedforward neural network; NB: Naïve bayes; LR: Logistic regression; DT: decision tree; SVM: Support vector machine; LASSO: Least absolute shrinkage and selection operator; KNN: K-nearest neighbor; RF: Random forest; ADB: AdaBoost; XGB: XGBoost).

Method	LSM-5 (Sec)	LSM-10 (Sec)	LSM-15 (Sec)	# of Models Trained	Total Time (Days)
DFNN	117.430	45.021	20.212	24,111	50.974
NB	0.060	0.046	0.026	18,109	0.028
LR	0.563	0.353	0.253	22,399	0.303
DT	0.048	0.037	0.032	107,351	0.145
LASSO	0.860	0.372	0.189	1024	0.017
SVM	12.197	2.876	0.362	1799	0.321
KNN	1.636	0.436	0.132	42,341	1.080
RF	0.774	0.603	0.549	27,000	0.602
ADB	0.655	0.508	0.403	13	0.000
XGB	4.710	4.566	3.850	46,980	7.137

## Data Availability

The data used in this study are available at datadryad.org (DOI 10. 5061/dryad.64964m0).

## Supplementary Materials

The following supporting information can be downloaded at: https://www.mdpi.com/article/10.3390/jcm11195772/s1, Table S1: The variables of the LSM datasets. Table S2: Description of the ML hyperparameters and Their Values Tested. Table S3: Significance test results: one-tailed (greater) Wilcoxon rank sum tests. Table S4: Significance test results: one-tailed (less) Wilcoxon rank sum tests.
