# Supplementary material for: Deep Learning and Machine Learning with Grid Search to Predict Later Occurrence of Breast Cancer Metastasis Using Clinical Data
Source: J Clin Med. 2022 Sep 29;11(19):5772. doi: 10.3390/jcm11195772 (PMC9570670; doi:10.3390/jcm11195772)
Supplement: Supplementary file 1 [file jcm-11-05772-s001.zip › jcm-1806678-supplementary.pdf]

Table S1. The variables of the LSM datasets

Variables included	Description	Values
race	race of patient	white, black, Asian, American Indian or Alaskan native, native Hawaiian or other Pacific islander
ethnicity	ethnicity of patient	not Hispanic, Hispanic
smoking	smoking history of patient	ex smoker, non smoker, cigarettes, chewing tobacco, cigar
alcohol usage	alcohol usage of patient	moderate, no use, use but nos (non otherwise specified), former user, heavy user
family history	family history of cancer	cancer, no cancer, breast cancer, other cancer, cancer but nos
age_at_diagnosis	age at diagnosis of the disease	0-49, 50-69, >69
menopausal_status	inferred menopausal status	pre, post
side	side of tumor	left, right
TNEG	triple negative status in terms of patient being ER, PR, and HER2 negative	yes, no
ER	estrogen receptor expression	neg, pos, low pos
ER_percent	percent of cell stain pos for ER receptors	0-20, 20-90, 90-100
PR	progesterone receptor expression	neg, pos, low pos
PR_percent	percent of cell stain pos for PR receptors	0-20, 20-90, 90-100
P53	whether P53 is mutated	neg, pos, low pos
HER2	HER2 expression	neg, pos
t_tnm_stage	prime tumor stage in TNM system	0, 1, 2, 3, 4, IS, 1mic, X
n_tnm_stage	# of nearby cancerous lymph nodes	0, 1, 2, 3, 4, X
stage	composite of size and # positive nodes	0, 1, 2, 3
lymph_nodes_removed	number of lymph nodes removed	0-11, 12-22, > 22
lymph_nodes_positive	number of positive lymph nodes	0, 1-8, >8
lymph_node_status	patient had any positive lymph nodes	neg, pos
histology	tumor histology	lobular, duct
size	size of tumor in mm	0-32, 32-70, >70
grade	grade of disease	1, 2, 3
invasive	whether tumor is invasive	yes, no
histology2	tumor histology subtypes	IDC, DCIS, ILC, NC

invasive_tumor_location	where invasive tumor is located	mixed duct and lobular, duct, lobular, none
DCIS_level	type of ductal carcinoma in situ	solid, apocrine, cribriform, dcis, comedo, papillary, micropapillary
re_excision	removal of an additional margin of tissue	yes, no
surgical_margins	whether residual tumor	res. tumor, no res. tumor, no primary site surgery
MRIs_60_surgery	MRIs within 60 days of surgery	yes, no

Table S2: Description of the ML hyperparameters and Their Values Tested (Notes: NB: Naïve bayes; LR: Logistic regression; DT: Decision tree; SVM: Support vector machine; LASSO: Least absolute shrinkage and selection operator; KNN: K-nearest neighbor; RF: Random forest; ADB: AdaBoost; XGB: XGBoost).

**Method**	**Hyperparameter**	**Description**	**Values**
Bernoulli NB	alpha	Laplace smoothing parameter	1,2,3, …, 499,500,700,1000
	fit_prior	Whether to learn class prior probabilities or not	True, False
	Binarize	The threshold value of the binarization of sample features.	0, 1, 2, 3, 4, 5
LR	C	Inverse of regularization strength (C=1/λ)	0.0001-10000(300 numbers distributed evenly on log scale)
	penalty	Used to specify the norm used in the penalization	'l2', 'none'
	solver	Algorithm to use in the optimization problem	'newton-cg', 'lbfgs', 'saga', 'sag'
DT	criterion	The function to measure the quality of a split	'gini', 'entropy'
	splitter	Used to control the randomness of dividing nodes in the decision tree	'best', 'random'
	max_depth	The maximum depth of the tree	3, 4, …, 31, 32
	max_features	Used to limit the number of features in the decision tree	'sqrt', 'log2', None

	min_samples_split	The minimum number of samples required to split an internal node	0.1, 0.2, 0.3, 0.4, 0.6, 0.8
	min_samples_leaf	The minimum number of samples required to be at a leaf node	1, 2
	class_weight	Used to set different weights for positive and negative samples in the case of unbalanced samples	None, 'balanced'
SVM	C	Inverse of regularization strength (C=1/λ)	$2^{-5}, 2^{-3}, \ldots, 2^{15}$
	gamma	Kernel coefficient	$2^{-15}, 2^{-11}, \ldots, 2^{3}$
	kernel	Specifies the kernel type to be used in the algorithm	'rbf', 'linear', 'sigmoid', 'poly'
	penalty	Used to specify the norm used in the penalization	l1
	solver	Algorithm to use in the optimization problem	'liblinear', 'saga'
KNN	n_neighbors	Number of neighbors	1, 2, …, 51, 100, 200, 300
	weights	Weights of each neighbor	'uniform', 'distance'
	algorithm	Algorithm used to compute the nearest neighbors	'kd_tree', 'brute'
	leaf_size	Leaf size passed to KDTree	10, 14, …, 38
	metric	The method of calculating tree distance	'euclidean', 'manhattan', 'chebyshev'
RF	n_estimators	The number of trees in the forest.	10, 50, 100, 200, 300
	criterion	Same as DT	'gini', 'entropy'
	min_samples_split	Same as DT	0.1, 0.2, 0.3, 0.4, 0.6, 0.8
	min_samples_leaf	Same as DT	1, 2
	max_depth	Same as DT	1, 2, …, 29, 30
	max_leaf_nodes	Same as DT	None
	max_features	Same as DT	'sqrt', 'log2', None
ADA	algorithm	The type of boosting algorithm	SAMME.R
	n_estimators	The maximum number of estimators at which boosting is terminated	100, 10
	learning_rate	Weight applied to each classifier at each boosting iteration	0.002
XGB	gamma	Minimum loss reduction required to make a further partition on a leaf node of the tree	0, 0.01, 0.1, 0.3, 0.5, 0.9

	learning_rate	Step size shrinkage used in update to prevents overfitting	0.01, 0.05, 0.1, 0.5, 0.9
	n_estimators	The number of boosting stages to perform	100
	subsample	The subsample is the sample proportion for the training	0.6, 0.7, 0.8, 0.9
	colsample_bytree	Colsample bytree is the sampling ratio of the feature	0.6, 0.7, 0.9
	max_depth	The maximum depth of the individual regression estimators	3, 4, …, 30, 31

Table S3: Significance test results: one-tailed (greater) Wilcoxon rank sum tests

X, Y		DNN, NB	DNN, LR	DNN, DT	DNN, LASSO	DNN, SVM	DNN, KNN	DNN, RF	ADB, DNN	DNN, XGB
5-year Metastasis	W	19	11	16	11	18	6	7	11	6
	p-value	0.111	0.655	0.274	0.655	0.155	0.925	0.889	0.655	0.925
	95 % CI	[-0.0174 Inf]	[-0.0269 Inf]	[-0.0112 Inf]	[-0.0254 Inf]	[-0.0057 Inf]	[-0.0453 Inf]	[-0.0463 Inf]	[-0.0388 Inf]	[-0.0520 Inf]
10-year Metastasis	W	10	19	19	22	22	12	8	14	5
	p-value	0.726	0.111	0.111	0.028	0.028	0.579	0.843	0.421	0.952

	95 % CI	[-0.0197 Inf]	[-0.0005 Inf]	[-0.0030 Inf]	[0.0012 Inf]	[0.0047 Inf]	[-0.0198 Inf]	[-0.0312 Inf]	[-0.0168 Inf]	[-0.0300 Inf]
15-year Metastasis	W	24	9	22	9	9	20	19	6	20
	p-value	0.011	0.799	0.030	0.799	0.799	0.071	0.104	0.929	0.071
	95 % CI	[0.0291 Inf]	[-0.0555 Inf]	[0.0013 Inf]	[-0.0505 Inf]	[-0.0529 Inf]	[-0.0012 Inf]	[-0.0026 Inf]	[-0.1120 Inf]	[-0.0017 Inf]

Table S4: Significance test results: one-tailed (less) Wilcoxon rank sum tests

X, Y		DNN, NB	DNN, LR	DNN, DT	DNN, LASSO	DNN, SVM	DNN, KNN	DNN, RF	ADB, DNN	DNN, XGB
5-year BCM	W	19	11	16	11	18	6	7	11	6
	p-value	0.925	0.421	0.790	0.421	0.889	0.111	0.155	0.421	0.111
	95 % CI	[-Inf 0.0583]	[-Inf 0.0252]	[-Inf 0.0380]	[-Inf 0.0241]	[-Inf 0.0735]	[-Inf 0.0145]	[-Inf 0.0032]	[-Inf 0.0223]	[-Inf 0.0041]
10-year BCM	W	10	19	19	22	22	12	8	14	5
	p-value	0.345	0.925	0.925	0.984	0.984	0.500	0.210	0.655	0.075

	95 % CI	[-Inf 0.0096]	[-Inf 0.0317]	[-Inf 0.0274]	[-Inf 0.0303]	[-Inf 0.0375]	[-Inf 0.0182]	[-Inf 0.0068]	[-Inf 0.0192]	[-Inf 0.0041]
15-year BCM	W	24	9	22	9	9	20	19	6	20
	p-value	0.994	0.265	0.982	0.265	0.265	0.953	0.929	0.104	0.953
	95 % CI	[-Inf 0.1494]	[-Inf 0.0826]	[-Inf 0.1431]	[-Inf 0.0745]	[-Inf 0.0719]	[-Inf 0.0927]	[-Inf 0.0920]	[-Inf 0.0149]	[-Inf 0.0979]
